# Data assimilation reveals behavioral dynamics of sea cucumbers as a model for slow-moving benthic animals

**DOI:** 10.1038/s41598-025-29171-3

**Published:** 2025-12-12

**Authors:** Tsutomu Takagi, Yuto Tanaka, Erica Sasano, Kouki Kanda, Yuichi Sakai

**Affiliations:** 1 Faculty of Fisheries Sciences, Hokkaido University, Sapporo, 060-0810 Japan; 2 Graduate School of Environmental Science, Hokkaido University, Sapporo, 060-0810 Japan; 3 Hakodate Fisheries Research Institute, Hakodate, 040-0051 Japan; 4 Present Address: Kansai Electric Power Co., Inc., Osaka, 530-8270 Japan; 5 Present Address: Organo Co., Inc., Tokyo, 136-8631 Japan

**Keywords:** Data assimilation, Sea cucumber, Fractal dimension, Movement ecology, Acoustic telemetry, Benthic organism tracking, Ecology, Mathematics and computing

## Abstract

Understanding the movement behavior of Japanese sea cucumbers (*Apostichopus japonicus*) is essential for ecological research and fisheries management. However, tracking their locomotion is challenging due to their slow movement and environmental variability. In this study, we employed acoustic telemetry combined with a data assimilation approach using the Kalman filter to estimate movement trajectories with high accuracy, overcoming the limitations of traditional visual tracking methods. To characterize movement complexity, we applied fractal dimension analysis, quantifying the randomness and variability of individual locomotion across different environmental conditions. Additionally, we examined the influence of key environmental factors, including water temperature, diel cycles, and boulder presence, using Generalized Linear Models (GLM). The results indicate that during the growing stage, higher water temperatures significantly increased movement activity, while boulder zones influenced movement differently depending on the season. This study also provides long-term tracking data on released sea cucumbers, offering new insights into their settlement and dispersal patterns. By combining acoustic telemetry, data assimilation, fractal analysis, and statistical modeling, we established a framework to investigate the behavioral dynamics of slow-moving benthic organisms. These findings enhance our understanding of sea cucumber ecology and provide a quantitative framework for future studies on marine invertebrate movement.

**Supplementary Information:**

The online version contains supplementary material available at 10.1038/s41598-025-29171-3.

## Introduction

The Japanese sea cucumber (*Apostichopus japonicus*), commonly known as “Manamako,” is widely distributed in coastal areas of Japan and has long been consumed as seafood. In China, dried sea cucumbers are highly valued as luxury food items. In 2020, *A. japonicus* accounted for approximately 8% (18.1 billion JPY) of the total export value of marine products from Japan, highlighting its significance as a key export commodity^1^. Given this context, there is growing concern that the increased market value of *A. japonicus* may drive greater fishing pressure on wild populations, particularly in Hokkaido^2^.

The production of *Manamako* seedlings has a history spanning more than 50 years, supported by a detailed production manual has been developed by the Hokkaido Sea Farming Experiment Station^3^. However, the postrelease detection rate of sea cucumbers has been reported to decrease significantly^4^, and the postrelease dynamics of sea cucumbers in natural habitats remain largely unknown. In particular, the intensity of dispersal and the survival rate of released individuals are still poorly understood.

To address these uncertainties, trials using microsatellite DNA markers for parent‒offspring identification have been conducted to evaluate the effectiveness of seedling release^5–7^. In one Hokkaido study, released sea cucumbers migrated up to 2,500 m from the release site within three years, and genetic diversity at the release site was similar to that in natural areas without seedling release.

Understanding the migratory behavior and environmental responses of *A. japonicus* is essential for effective resource management. However, long-term monitoring is challenging because of the reclusive nature of sea cucumbers and difficulties in tag attachment due to their lack of hard tissues^8^. Physical tag attachment often results in high dropout rates, as shown in the orange-footed sea cucumber (*Cucumaria frondosa*) and the California sea cucumber (*Parastichopus californicus*)^9,10^. Some studies demonstrated that single spaghetti tags can achieve relatively high retention rates under certain conditions^8,11^, while others reported poor retention for passive induced transponder (PIT) tags in tropical species^12^. However, Gianasi et al. reported that embedding PIT tags through oral tentacles was effective in the commercial species *C. frondosa*^13^. These findings suggest that tag retention rates and suitable attachment methods vary among species and environments.

Sea cucumbers are often considered sedentary benthic organisms^14–16^, yet some species can disperse over notable distances^17^, and others, such as *A. japonicus*, show seasonal behaviors like aestivation during which activity is reduced^18^. Laboratory studies have shown that high flow velocity reduces locomotion speed in sea cucumbers^19–21^, while field and experimental work revealed that active buoyancy adjustment (ABA) can enable much faster movement than benthic crawling^22^. Tropical species have been observed to travel several meters per day and, in some cases, over 100 m in the long term^23^.

Given these findings and the possibility of high-speed movement, it may be necessary to investigate individual movements at fine temporal scales. Small acoustic tags can estimate individual locations, but very few studies have applied them to evaluate long-term movement in sea cucumbers.

In this study, we developed a method to track the movements of wild *A. japonicus* along the southern coast of Hokkaido, Japan, by attaching ultrasonic transmitter tags and using multiple acoustic receivers. The aim of this study was to focus on relatively enclosed coastal areas in Hokkaido, which are representative of the hydrodynamically sheltered environments favored for the release of individuals. By conducting long-term monitoring of released individuals in such sheltered waters, we sought to clarify the behavioral characteristics of this species under relatively calm hydrodynamic conditions, thereby providing insights relevant to the survival, production, and management of sea cucumbers released in enclosed coastal environments.

We first propose an analytical approach to estimate the movement trajectories of slow-moving marine benthic echinoderms, such as sea cucumbers, via small ultrasonic transmitters and data assimilation techniques. Whereas activity is reduced during the resting stage, it increases during the winter growth period^24^. In addition, diel activity patterns in this species tend to be higher at night, with individuals showing increased emergence from shelters after sunset under various photoperiod conditions^25^. These seasonal and diel patterns help interpret the movement data collected in this study. Using this approach, we investigated the effects of environmental factors—specifically seasonal changes and water temperature—on the behavior of this species. Furthermore, to quantify differences in planar movement trajectories among individuals and across seasons, we calculated the fractal dimension of the movement paths. Our results indicate that variation in fractal dimension may provide a useful metric for assessing behavioral differences in this species.

## Materials and methods

### Position identification of individuals via ultrasonic transmitters

In this study, we used ultrasonic transmitters (V5-1 H, VEMCO Co., Ltd.) and stationary ultrasonic receivers (VR2W, VEMCO Co., Ltd.) to identify the positions of individual sea cucumbers. The V5 transmitters operated at an acoustic frequency of 180 kHz. The transmitter measures 5.6 mm in diameter and 12.7 mm in length, with a weight of 0.65 g in air and 0.38 g in water, and has a transmission range of 100–150 m. The transmission period is programmed to automatically stop after 40 or 70 days to prevent interference with other transmitters in case of loss. Each transmitter has a unique ID code and transmits signals randomly at intervals of 90–150 s.

The receiver measures 73 mm in diameter and 308 mm in length, weighing 1190 g in air and 50 g in water. It records the ID codes received from transmitters along with the time of reception in its internal memory. The maximum storage capacity of the receiver is approximately 16 million detections, and the recorded temporal resolution is 1 millisecond (1/1000 second).

The two-dimensional coordinates of individual positions were determined by deploying multiple ultrasonic receivers on the seabed and calculating the positions of the transmitters on the basis of the time differences in the ultrasonic signals received by the receivers. Since the position of a transmitter lies on a hyperbola defined by two receivers as focal points, the intersection of hyperbolas generated by multiple pairs of receivers was estimated as the transmitter’s position. Positions were estimated only when signals were detected by all receivers, ensuring that differences in the number of detecting receivers did not affect the results.

Espinoza et al. evaluated the accuracy and performance of the Vemco positioning system (VPS), which applies this principle, in a controlled basin^26^. They reported that the distance error between the estimated and actual positions of a fixed transmitter within the receiver array was 2.13 ± 1.3 m. Additionally, they reported that depth did not affect positional accuracy. Nanami et al. applied this method to estimate the home range size of the coral reef grouper (*Epinephelus ongus*)^27^.

### Experimental site and setup

In Hokkaido, a proposal to release and cultivate *A. japonicus* in harbors is considered a means to improve the functionality of underutilized fishing ports. In this study, we investigated the behavioral characteristics of this species when released into a fishing port.

**Fig. 1:**
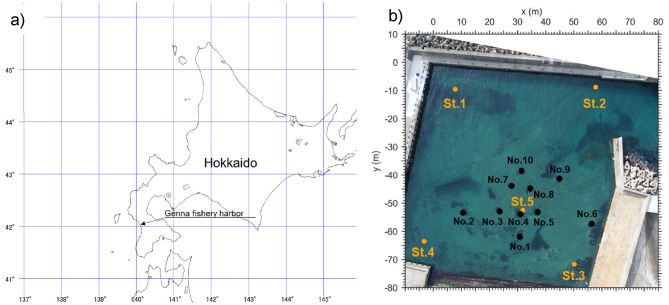
(**a**) Map of Genna Fishing Port in Otobe-cho, Hokkaido, Japan. (**b**) The  (orange) circle represents the receiver, while the • (black) circle represents a boulder. The fixed synchronized tag was installed at the same location as receiver Station 5 (St. 5).

The experimental site was Genna Fishing Port, which is located in Otobe-cho, Nishi-gun, Hokkaido (Fig. 1a). The Genna Fishing Port is an active port, with its entrance remaining open. The port is nearly rectangular in shape, with each side measuring up to 80 m. To determine the positional coordinates of the sea cucumbers, a two-dimensional horizontal coordinate system (*x*‒*y*) was established, as shown in Fig. 1b.

During the experimental period, divers regularly monitored tagged *A. japonicus* individuals. All tagged individuals were observed on the seafloor, with no cases of floating in the water column. Individuals were often found within spaces between boulders, but not prominently on top of them. Acoustic receivers were mounted approximately 1 m above the seafloor. Given the time-difference-of-arrival method and the 1/1000 s clock precision of the receivers, this height corresponds to a spatial resolution of ~ 1 m; at typical distances of ≥ 20 m from the nearest receiver, such a vertical offset is negligible. The boulders were ≤ 0.5 m in height, so even if an individual was located on top of a boulder, the resulting vertical displacement would still be within the measurement error. Therefore, movement analysis was conducted in the horizontal (x–y) plane.

The experimental periods were from October 9 to November 11, 2020, and from February 22 to April 2, 2021. Tanaka divided the life cycle of sea cucumbers into five stages: the resting stage, recovery stage, growing stage, mature stage, and shedding stage^24^. According to his report, the resting stage occurs from October to November, whereas the growing stage occurs from February to April, suggesting that behavioral differences may arise depending on the time of year.

### Receiver installation and synchronization

The receivers were installed at a height of approximately 1 m above the seabed. As shown in Fig. 1b, receivers St. 1 to St. 5 were placed at the corners of the port and near the release point. Prior to the experiment, in situ range tests were conducted to verify detection conditions. A diver carried a V5 transmitter throughout the port in multiple directions and at various distances (all ≤ 100 m from a receiver), and continuous detections were confirmed across the study area. Since the internal clocks of the receivers operated independently, clock synchronization was necessary. To achieve this, a transmitter for time synchronization (hereafter referred to as the “synchronized tag”) was directly attached to the receiver at St. 5, which was used as the reference for clock synchronization. The synchronized tag had the same specifications as the other transmitters, emitting signals randomly at intervals of 90–150 s.

**Fig. 2:**
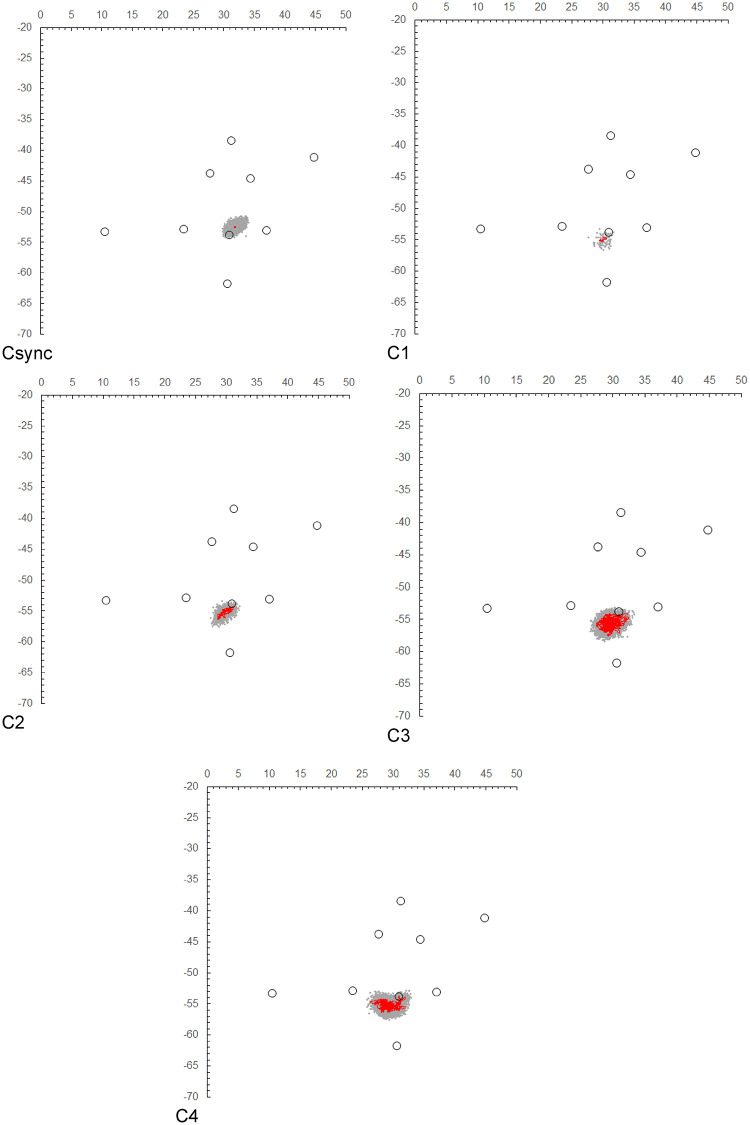
Position estimation plots ( gray dots) and movement trajectory estimations ( red lines) obtained using a random walk model-based Kalman filter (with smoothing), derived from the hyperbolic curves of the synchronized tags in C and individuals C1–C4. C1 represents the movement trajectory estimation from October 9, 2020, to October 19, 2020, C2 from October 9, 2020, to October 15, 2020, and C3, C4, and the synchronized tags (Csync) represent the trajectory estimations for the period from October 9, 2020, to November 11, 2020. The 〇 white circles indicate the centroid positions of the artificial boulder zones.

**Fig. 3:**
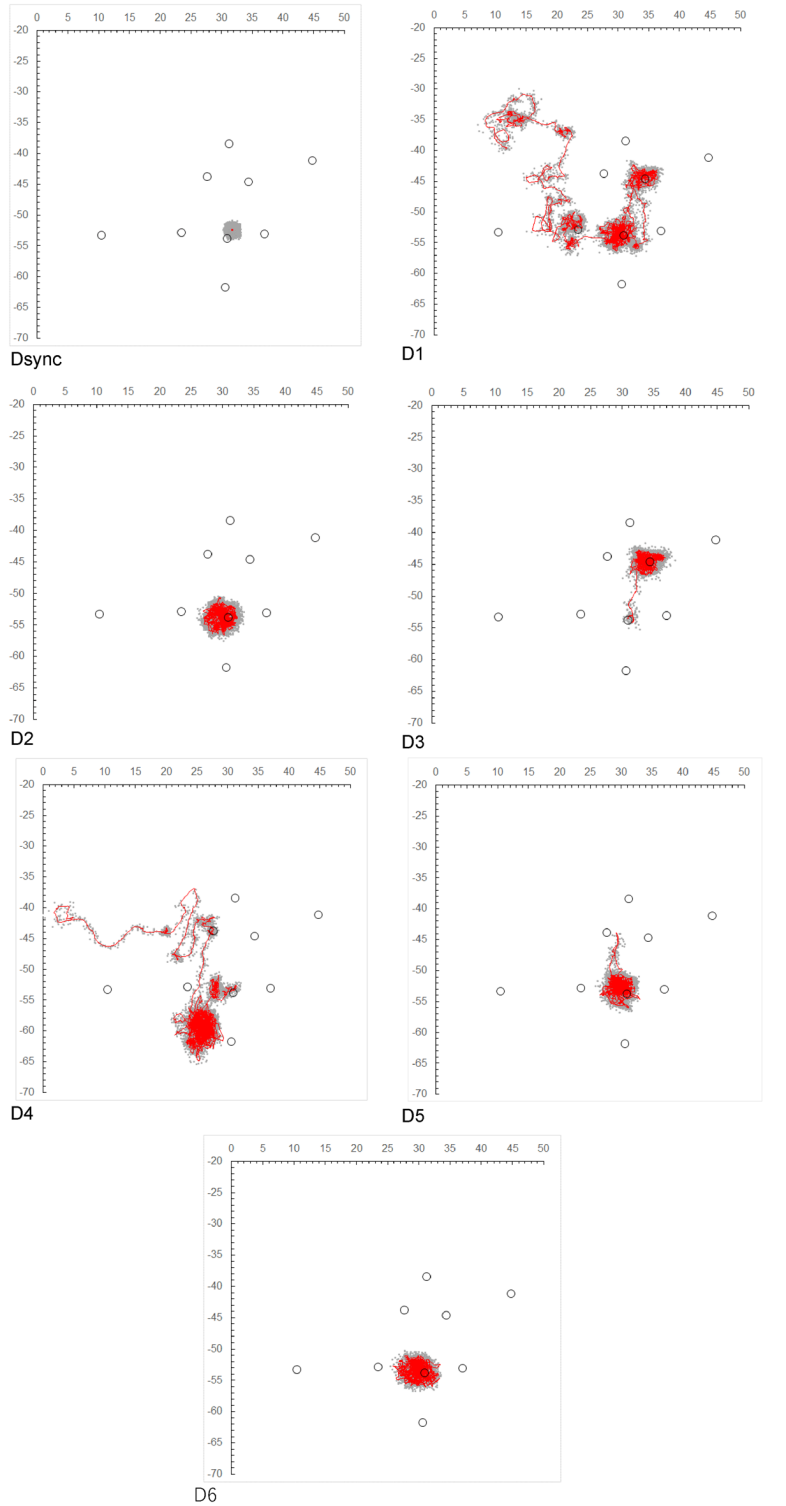
Position estimation plots ( gray dots) and movement trajectory estimations ( red lines) obtained using a random walk model-based Kalman filter, derived from the hyperbolic curves of the synchronized tags in D and individuals D1–D6. D1–D4, D6, and the synchronized tag (Dsync) show movement trajectory estimations for the period from February 22, 2021, to April 2, 2021, while D5 shows estimations for the period from February 22, 2021, to March 31, 2022. The 〇 white circles indicate the centroid positions of artificially placed boulders.

In addition, fixed synchronized tags were deployed within the receiver array during both the C-group and D-group experiments. Their positions were estimated by hyperbolic positioning and compared with the same positions after Kalman filter smoothing (see Figs. 2 and 3). The dispersion of the unsmoothed positions around the known fixed locations was used as an empirical measure of local positioning error.

To further quantify the positioning accuracy, we calculated the horizontal distance between the true location of the synchronized tag (measured using a laser 3D rangefinder) and the estimated positions obtained (1) by hyperbolic positioning and (2) after Kalman smoothing. The distribution of localization errors for each method was summarized as box plots (Supplementary Fig. S1). These calculations allow a direct comparison between the raw and smoothed estimates in terms of localization accuracy.

The positions of the receivers were mapped in two-dimensional horizontal plane coordinates via a laser 3D rangefinder (DISTO90, Leica Co., Ltd.). A diver held a marker directly above each receiver installation site at the sea surface, and the positions of these markers were measured with the rangefinder. The average water temperature during the experimental periods was 16.4 °C from October 9 to November 11, 2020, and 7.6 °C from February 22 to April 2, 2021.

In addition, to monitor water-level fluctuations throughout the experimental period, a pressure-based depth recorder (WAVE HUNTER08-ΣWH-403, IOTechnic Co., Ltd., Japan) was installed near the center of the receiver array at a fixed height above the seabed. Continuous depth records were used to evaluate potential tidal and diel (morning–evening) variations in water level that could influence acoustic propagation conditions.

### Experimental individuals

In this study, *A. japonicus* collected from the experimental site, Genna Fishing Port, were used. The wet weights of the individuals used in the experiment are shown in Table 1.

**Table 1:** Wet weights (gf) of experimental individuals, their release dates, and tracking durations (days).

ID	Release date	Tracking duration (days)	Wet weight (gf)
C1	Oct/9/2020	11	198
C2	Oct/9/2020	7	166
C3	Oct/9/2020	34	191
C4	Oct/9/2020	34	192
D1	Feb/22/2021	40	257
D2	Feb/22/2021	40	214
D3	Feb/22/2021	40	216
D4	Feb/22/2021	40	191
D5	Feb/22/2021	38	195
D6	Feb/22/2021	40	210

It has traditionally been considered difficult to attach tags to *A. japonicus* because of their lack of hard tissue. However, studies on tag retention have been conducted by Fujino et al. and Furukawa et al. ^8,11^. In this study, the transmitter was externally attached to individuals via a minimally invasive method developed by Sakai et al. (presented at the Fisheries Society of Japan). This attachment technique, specifically designed for sea cucumbers lacking hard tissue, has been reported to ensure high tag retention without affecting behavior or health^28^.

The bottom sediment in the fishing port at the experimental site consisted mainly of sandy mud, with patches of seagrass. To evaluate the effects of substrate differences on behavior, 10 artificial boulder zones (No. 1–No. 10) were established (Fig. 1b). The boulder zone at No. 4 was designated the release point, and behavior was tracked from this area.

All individuals equipped with transmitters were released at the designated release point, and their subsequent behaviors were monitored. Since transmitters can potentially detach from sea cucumbers, researchers inspect their attachment every 1–3 weeks after release by diving observation. In this study, only the periods during which transmitter attachment was confirmed were analyzed, and individuals whose transmitters could not be confirmed were excluded from the analysis.

### Estimation of movement trajectories via a Kalman filter

Sibert et al. analyzed the paths of bigeye tuna (*Thunnus obesus*) equipped with archival tags by describing the equation of state as a random walk^29^. In their study, the movement of individuals was represented via the following state-space model and estimated with a Kalman filter:

1 $$\:{\varvec{x}}_{t}={\varvec{x}}_{t-1}+{\varvec{v}}_{t}$$

2 $$\:{\varvec{y}}_{t}={\varvec{x}}_{t}+{\varvec{w}}_{\varvec{t}}$$

Here, ***x***_*t*_ is the state vector in the system model expressed in Eq. (1), representing the position coordinates of the individual in the *x*-*y* two-dimensional plane. ***y***_***t***_​ is the observation vector in the observation equation expressed in Eq. (2). Both ***v***_***t***_​ and ***w***_***t***_​ are Gaussian white noise, given as ***v***_*t*_∼*N*(0, ***Q***_*t*_) and ***w***_***t***_∼*N*(0, ***R***_*t*_), where ***Q***_*t*_ and ***R***_*t*_​ represent the variance‒covariance matrices. Equation (1) indicates that the movement of an individual is predicted via a random walk.

In this study, the movement of sea cucumber was predicted via Eq. (1) via the Kalman filter. The predicted position was iteratively refined by filtering using the observed values of ***y***_***t***_.

In addition to the random walk model in Eq. (1), this study also considered a system model that incorporates the movement speed of individuals and evaluated its applicability. The algorithm of the Kalman filter is shown in the following Eq. 

3 $$\:{\varvec{x}}_{t}={\varvec{F}}_{t}{\varvec{x}}_{t-1}+{\varvec{G}}_{t}{\varvec{v}}_{t}$$

4 $$\:{\varvec{y}}_{t}={\varvec{H}}_{t}{\varvec{x}}_{\varvec{t}}+{\varvec{w}}_{t}$$

5 $$\:{\varvec{x}}_{t|t-1}={\varvec{F}}_{\varvec{t}}{\varvec{x}}_{t-1|t-1}$$

6 $$\:{\varvec{V}}_{t|t-1}={\varvec{F}}_{t}{\varvec{V}}_{t-1|t-1}{\varvec{F}}_{t}^{T}+{\varvec{G}}_{t}{\varvec{Q}}_{t}{\varvec{G}}_{t}^{T}$$

7 $$\:{\varvec{K}}_{t}={\varvec{V}}_{t|t-1}{\varvec{H}}_{t}^{T}{\left({\varvec{H}}_{t}{\varvec{V}}_{t|t-1}{\varvec{H}}_{t}^{T}+{\varvec{R}}_{t}\right)}^{-1}$$

8 $$\:{\varvec{x}}_{t|t}={\varvec{x}}_{t|t-1}+{\varvec{K}}_{t}\left({\varvec{y}}_{t}-{\varvec{H}}_{t}{\varvec{x}}_{t|t-1}\right)$$

9 $$\:{\varvec{V}}_{t|t}={\varvec{V}}_{t|t-1}-{\varvec{K}}_{t}{\varvec{H}}_{t}{\varvec{V}}_{t|t-1}$$

Here, ***x***_*t*_ is the state vector, ***F***_*t*_ is the transition matrix of the system model, and ***v***_*t*_ is the system noise, which follows a multivariate normal distribution with a mean vector of 0 and a covariance matrix Σ = σ²***I***. ***H***_*t*_ represents the transformation matrix that relates the observation vector to the state vector, whereas ***G***_*t*_​ represents the transformation matrix that relates the system noise to the state vector. In subscripts such as *t*∣*t* − 1, the first part (*t*) corresponds to the time of the state, and the second part (*t* − 1) represents the most recent time of the observed data.

Let ***x***_*t*_ be the estimated two-dimensional position vector in the plane at time t for an individual, expressed as ***x***_***t***_=(*x*_*t*_, *y*_*t*_)^*T*^, and let ***y***_*t*_ ​ be the observed vector at time t, expressed as ***y***_t_=(*x*_*m, t*_, *y*_*m, t*_)^*T*^. Here, ***w***_*t*_ represents observation noise, which follows a multivariate normal distribution with a mean vector of **0** and a covariance matrix Σ = τ²***I***. When ***F***_*t*_​, ***H***_*t*_ ​, and ***G***_*t*_ are identity matrices, Eqs. (3) and (4) represent a state-space model where the individual behavior follows a random walk, as described in Eqs. (1) and (2).

Under this model, the Kalman filter is used to estimate the individual position ***x***_*t*∣*t*_ at time *t*, along with the variance-covariance matrix ***V***_*t*∣*t*_​, which quantifies the uncertainty in the estimation.

Equations (5) and (6) describe the prediction step, where the predicted position ***x***_*t*∣*t*−1_ and its variance covariance ***V***_*t*∣*t−1*_​ at time t are estimated one step ahead, given the estimated position and variance covariance of the state at time *t* − 1.

Equations (8) and (9) represent the filtering step, in which the predicted position ***x***_*t*∣*t*−1_ and its variance covariance ***V***_*t*∣*t*−1_, estimated in the prediction step, are updated using the observed values ***y***_*t*_ obtained at time *t*. The Kalman gain ***K***_*t*_​, used in Eqs. (8) and (9), is defined in Eq. (7)^30^.

The noise in the state-space representation of the model in this study was estimated by maximum likelihood from the observed values.

Fixed-interval smoothing via the Kalman filter enhances the accuracy of filtering by incorporating all the observed data. Using Rauch et al.‘s method (Kalman smoother), the state estimates obtained from Eqs. (8) and (9) were further smoothed^31^.

10 $$\:{\varvec{x}}_{t|T}={\varvec{x}}_{t|t}+{\varvec{A}}_{t}\left({\varvec{x}}_{t+1|T}-{\varvec{x}}_{t+1|t}\right)$$

11 $$\:{{\varvec{V}}_{t|T}={\varvec{V}}_{t|t}+{\varvec{A}}_{t}\left({\varvec{V}}_{t+1|T}-{\varvec{V}}_{t+1|t}\right)\varvec{A}}_{t}^{T}$$

12 $$\:{\varvec{A}}_{\varvec{t}}={\varvec{V}}_{\varvec{t}|\varvec{t}}{\varvec{F}}_{\varvec{t}+1}^{\varvec{T}}{\varvec{V}}_{\varvec{t}+1|\varvec{t}}^{-1}$$

Here, *t* = *T* − 1, …, 0. By providing the initial conditions ***x***_*T*∣*T*_​ and ***V***_*T*∣*T*_ ​, the formulations (10)–(12) are applied sequentially to compute ***x***_*T*−1∣*T*_, ***x***_*T*−2∣*T*_…, ***x***_0∣*T*_ and ***V***_*T*−1∣*T*_, ***V***_***T***−2∣*T*_…, ***V***_0∣*T*_​.

Note that ***x***_*T*∣*T*_ and ***V***_*T*∣*T*_ are the values estimated by the Kalman filter at *t* = *T*. The terms ***x***_*t*+1∣*t*_, ***V***_***t***+1∣*t*_​, ***x***_*t*∣*t*_​, and ***V***_***t***∣*t*_​ on the right-hand side are obtained during the Kalman filter calculation process^30^.

For the *x*-coordinate of an individual’s position, the coordinate after a small time increment Δ*t* can be expressed via a Taylor expansion as^32^:

13 $$\:x\left(t+\varDelta\:t\right)=x\left(t\right)+\varDelta\:t\frac{dx\left(t\right)}{dt}+o(\varDelta\:{t}^{2})$$

The following equation can be approximated:

14 $$\:x\left(t+\varDelta\:t\right)\cong\:x\left(t\right)+\varDelta\:t\frac{dx\left(t\right)}{dt}=x\left(t\right)+u\left(t\right)\varDelta\:t$$

where $\:u\left(t\right)=\frac{dx\left(t\right)}{dt}$ represents the movement velocity of the individual at time *t*.

By applying the same procedure to the y-coordinate component of the individual’s position, the state vector ***x***_*t*_ and the transition matrix ***F***t ​in Eq. (3) can be expressed as follows.

15 $$\:{\varvec{x}}_{\varvec{t}}={\left({x}_{t},\:{y}_{t},\:{u}_{t},\:{v}_{t}\right)}^{\varvec{T}}$$

16 $$\:{\varvec{F}}_{t}=\left(\begin{array}{cccc}1&\:0&\:\varDelta\:t&\:0\\\:0&\:1&\:0&\:\varDelta\:t\\\:0&\:0&\:1&\:0\\\:0&\:0&\:0&\:1\end{array}\right)$$

Here, *u*_*t*_​ and *v*_*t*_ represent the *x*- and *y*-components of the individual’s velocity at time *t*. Δ*t* in Eq. (16) is the time interval between t and *t* − 1, which is assumed to be sufficiently small. If both the position vector (*x*, *y*)^*T*^ and the velocity vector (*u*, *v*)^*T*^ of the individual can be observed as ***y***_***t***_=(*x*_*m, t*_, *y*_*m, t*_, *u*_*m, t*_, *v*_*m, t*_)^*T*^, then ***H***_*t*_​ can be expressed as follows.

17 $$\:{H}_{t}=\left(\begin{array}{cccc}1&\:0&\:0&\:0\\\:0&\:1&\:0&\:0\\\:0&\:0&\:1&\:0\\\:0&\:0&\:0&\:1\end{array}\right)$$

This formulation enables the velocity vector of each individual to be estimated from the observed position information$\:{\varvec{y}}_{\varvec{t}}={\left({x}_{mt},{y}_{mt}\right)}^{T}$. The Kalman smoother fixed-interval smoothing formulation (10)–(12) can similarly enhance the filtering accuracy for the state estimate $\:{\varvec{x}}_{\varvec{t}}={\left({x}_{t},\:{y}_{t},\:{u}_{t},\:{v}_{t}\right)}^{\varvec{T}}$.

As previously described, formulations (10)–(12) allow $\:{\varvec{x}}_{T-1|T},{\varvec{x}}_{T-2|T},\:\dots\:,\:{\varvec{x}}_{0|T}$ and $\:{\varvec{V}}_{T-1|T},{\varvec{V}}_{T-2|T},\:\dots\:,{\varvec{V}}_{0|T}$ to be computed sequentially.

### Estimation of system noise *v*_*t*_ and observation noise *w*_*t*_

To estimate the position coordinates of an individual via the Kalman filter algorithm, it is necessary to first estimate the system noise ***v***_*t*_ and the observation noise ***w***_*t*_​ in the state Eq. (3) and the observation Eq. (4).

Now, let the time series data up to time ***T*** (> 1) be **Y**_*t*_ = (***y***_1_…, ***y***_*t*_). In this case, the likelihood *L*(*θ*) of the state-space model defined by Eqs. (3) and (4) is given as:

18 $$L(\theta)= p(\boldsymbol{Y}_{T} \mid \theta)= \int p(\boldsymbol{x}_{0})  \prod_{t=1}^{T}    p(\boldsymbol{y}_{t} \mid \boldsymbol{x}_{t}, \theta)    p(\boldsymbol{x}_{t} \mid \boldsymbol{x}_{t-1}, \theta)    d\boldsymbol{X}_{T}$$

​Here, ***θ*** represents the unknown parameters, where *θ*=(σ, τ), and σ and τ are the standard deviations of the system noise and observation noise, respectively. These parameters can be numerically estimated by maximizing the likelihood *L*(***θ***) via maximum likelihood estimation^33^. Note that ***X***_*T*_=(***x***_0_, ***x***_1,_ ., ***x***_*T*_).

In this study, the dlm package^34^ (Petris, 2010) in R (R Development Core Team, 2021)^35^ was used to estimate the unknown parameters ***θ***. To maximize the likelihood function for estimating the unknown parameters, the L-BFGS-B algorithm, a variant of the quasi-Newton method, was applied^36^.

Since σ is defined by ***Q***_t_ ​, which constitutes ***v***_*t*_​ in the system model (Eq. 3), it can serve as an indicator of the magnitude of an individual’s movement within a unit time. Comparing the magnitude of σ allows for the comparison of movement sizes between individuals.

On the other hand, τ is defined by ***R***_*t*_ ​, which constitutes ***w***_*t*_​ in the observation model (Eq. (4)) and represents the level of noise in the observation system. If the same observation system is used, the magnitude of τ is expected to be similar.

Therefore, we compared the values of σ derived from the observed data of individuals C and D to investigate whether differences in movement exist between individuals or across seasons.

One of the authors, Sakai released three individual sea cucumbers equipped with Vemco V5 ultrasonic transmitter tags into a flume tank. The distance traveled and movement speed of the tagged individuals were measured for 350 h and compared with those of nontagged individuals^37^. The results revealed no significant differences in distance traveled or movement speed between the tagged and nontagged individuals.

### Estimation of fractal dimension

Mandelbrot generalized Richardson’s observation that the length of a border varies with the scale of the map used and that the scale and border length exhibit a linear correlation when plotted as logarithms. He named figures with this characteristic “fractals”^38^. The fractal dimension increases as the figure is composed of more complex, jagged lines, such as a coastline, and decreases, approaching 1, as the figure consists of smoother curves.

Sea cucumbers move along the seafloor over time, making it possible to estimate the fractal dimension of their movement trajectories. Since the trajectory of an individual exhibits a specific fractal dimension, comparing the fractal dimensions of movement trajectories among different individuals and seasons may reveal certain trends.

The box-counting method is a commonly used approach to approximate the fractal dimension of general figures^39^. Higuchi conceptualized time series data as patterns of line segments that fill a plane and quantified the complexity of these patterns via fractal dimensions^40^.

When *L*(Δ*t*) represents the length of the time series data coarse-grained at scale Δ*t*, the time series *X*(t) is defined as fractal if the graph of log(Δ*t*) vs. log(*L*(Δ*t*)) forms a straight line. Coarse-graining the time series data *X*(t) at a scale Δ*t* = *k* for *t* = 1,2, ,…, *N*, the time series dataset can be expressed for each sampling start time *m* = 1, 2,…, *k* as follows:

19 $$\begin{aligned} {\tilde {X}_1}\left( k \right); & X\left( 1 \right),~X\left( {1+k} \right),~X\left( {1+2k} \right), \cdots \\ \vdots \\ {\tilde {X}_m}\left( k \right); & X\left( m \right),~X\left( {m+k} \right),~X\left( {m+2k} \right), \cdots \\  \vdots \\{\tilde {X}_k}\left( k \right); & X\left( k \right),~X\left( {k+k} \right),~X\left( {k+2k} \right), \cdots. \end{aligned}$$

From above, the coarse-grained length *L*_*m*_(k) for each time series set $\:{\stackrel{\sim}{X}}_{m}\left(k\right)$ is given by:

20 $${L_m}\left( k \right)=\left\{ {\left( {\mathop \sum \limits_{{i=1}}^{{\left[ {\frac{{N - m}}{k}} \right]}} \left\| {X\left( {m+ik} \right) - X\left( {m+\left( {i - 1} \right) \cdot k} \right)} \right\|} \right)\frac{{N - 1}}{{\left[ {\frac{{N - m}}{k}} \right] \cdot k}}} \right\}/k$$

Here, [⋅] denotes the floor function, and ∥⋅∥ represents the Euclidean norm.

The average length <*L*(k)> over all *k* time series sets *L*_*m*_(*k*) coarse-grained at scale *k* is then defined as:

21 $$\left\langle {L\left( k \right)} \right\rangle =\frac{{\mathop \sum \nolimits_{{m=1}}^{k} {L_m}\left( k \right)}}{k}$$

If the graph of (log_2_(*k*), log_2_(<*L*(*k*)>) forms a straight line, the time series data are defined as fractal. The slope of the graph, −*D*, represents the fractal dimension *D*.

Since *D* reflects how the plane is covered, it can take any real value between 1 ≤ *D* ≤ 2 ^40^.

In this study, the fractal dimension *D* of the movement trajectory of an individual with a transmitter in the *x*‒*y* plane was estimated via Higuchi’s method described above. On a relatively smooth coastline, the fractal dimension is close to 1, the dimension of a line, whereas on the west coast of Britain, it is slightly larger, at 1.25^38^. A fractal dimension of *D* = 2 represents a time series that nearly fills the graph. The value of *D* indicates the complexity of the figure.

The time series data of movement trajectories used to calculate the fractal dimension consisted of hourly estimated positions. The data for fractal dimension analysis during the observation period included 100 consecutive data points (100 h). Time series datasets with gaps of more than one consecutive hour, due to missing signals, were excluded from the analysis. For comparison, the fractal dimension was also calculated for the time series data obtained from the position estimation of fixed synchronized tags. Individual C1 was excluded from the fractal dimension analysis because it frequently had long gaps in its measurements, making it impossible to obtain 100 consecutive movement trajectory points.

### Statistical analysis

#### Evaluation of differences in fractal dimensions

The slope of the line in the graph of (log_2_*k*, log_2_<*L*(*k*)>) represents the fractal dimension. The fractal dimension of the figures derived from the movement trajectories of different individuals was evaluated by comparing the slopes of their respective regression lines. A test for the parallelism of the regression lines was conducted to determine whether the fractal dimensions were significantly different.

The parallelism of the two regression lines was assessed via the F value obtained via the following equation:

22 $$\:F=\frac{{(S}_{1}-{S}_{2})/{(\phi\:}_{A}-{\phi\:}_{B})}{{S}_{2}/{\phi\:}_{B}}$$

Here, *S*_1_​ represents the total sum of the residual squares between the plot points of each group and the regression line when the slopes of the regression lines for the two groups are assumed to be identical. Conversely, *S*_2_​ is the total sum of the residual squares between the plot points of each group and the regression line when the slopes of the regression lines for the two groups are treated separately.

The degrees of freedom, *ϕ*_A_​, are calculated as the difference between the total number of data points under consideration and the number of parameters (3) for the two regression lines with a common slope. Similarly, *ϕ*_*B*_​ is calculated as the difference between the total number of data points and the number of parameters (4) for the two regression lines obtained separately.

The calculated *F* value was compared with *F*(1, *n* − 4, *p* = 0.01) at the 1% level of significance to evaluate whether the two regression lines are parallel.

#### Comparison of positional displacement

The positional displacement of individuals was evaluated via the Wilcoxon rank sum test with continuity correction to determine whether it differed by release period and whether there were differences in proximity to the boulder zone.

#### Assessment of factors affecting the positional displacement of individuals

To investigate how water temperature at the release point, time of day (daytime vs. nighttime), and proximity to the boulder zone influence the hourly positional displacement of individuals, a multiple regression analysis using generalized linear models (GLMs) was conducted in the R statistical environment^35^ (R Development Core Team, 2021). The response variable was the hourly positional displacement distance (dt_1_), whereas the explanatory variables included water temperature, time of day (daytime or nighttime), and proximity to the boulder zone. Water temperature was treated as a continuous variable, whereas time of day and proximity to the boulder zone were treated as categorical variables.

The link function for the applied GLM was logarithmic, and the probability distribution (error structure) of dt_1_ was assumed to follow a gamma distribution. The GLM is summarized by the following equation:

23 $$Y_{i} \sim Gamma\left( {\alpha ,s_{i} } \right)$$

24 $$\:E\left[{Y}_{i}\right]=\alpha\:{s}_{i}=\mu\:$$

25 $$\:\text{log}\left({\mu\:}_{i}\right)={\beta\:}_{0}+{\beta\:}_{1}{X}_{1i}+{\beta\:}_{2}{X}_{2i}+{\beta\:}_{3}{X}_{3i}$$

Here, Yi​ is the response variable representing the hourly positional displacement (dt_1_), *X*_1*i*_ ​ represents the water temperature, *X*_2*i*_ is a categorical variable indicating proximity to the boulder zone (1 if within the zone, 0 otherwise), and *X*_3*i*_ is a categorical variable representing the time of day (1 for daytime, 0 for nighttime). Proximity to the boulder zone was defined as being within a 2-meter radius of the centroid of the boulder zone.

*β*_0_​ is the intercept of the regression model, whereas *β*_1_, *β*_2_, and *β*_3_​ are the regression coefficients. Additionally, *α* is the shape parameter of the gamma distribution, which is constant, and *s*_*i*_ is the scale parameter that varies according to the changes in the linear predictor *µ*.

Prior to model fitting, we assessed potential collinearity between water temperature and time-of-day using the Variance Inflation Factor (VIF). The VIF values for both variables were approximately 1.01 in both groups, far below the threshold of 2 commonly used to indicate problematic collinearity, confirming that these predictors were statistically independent.

## Results

### Trajectory plots for individuals C and D

The trajectory plot points for Individual C and Individual D, obtained from the intersection of the hyperbolic curves, are shown in Figs. 2 and 3, respectively. The plot points of the C-synchronized and D-synchronized tags are also included. Additionally, the state estimates $\:{\varvec{x}}_{t|t}$ for each individual, derived from the Kalman filter based on the formulation of Eq. (3) to (12), are displayed in the figures. The system model was assumed to be a random walk model, as described in Eq. (1). If the observation vector $\:{{\varvec{y}}_{\varvec{t}}=\left({x}_{m,t},{y}_{m,t}\right)}^{\varvec{T}}$ at time *t* was missing (i.e., NA), instead of imputing the previous observation vector ***y***_*t*−1_, the prediction step (***x***_*t*∣*t*−1_,***V***_*t*∣*t*−1_) was executed as usual, and the filtering step was skipped. Consequently, the filtering distribution at time *t* was set equal to the one-step-ahead predictive distribution from time *t* − 1, i.e., ***x***_*t*∣*t*_=***x***_*t*∣*t*−1​_ and ***V***_*t*∣*t*_=***V***_*t*∣t−1_​, until the next observation became available.

Figure 2 shows that for Individual C, including the synchronized tags, the position estimation plots obtained from the hyperbolic curves are scattered and significantly spread. However, the one-month location estimates for the synchronized tag (C_sync) using the Kalman filter demonstrate almost pinpoint accuracy, effectively illustrating that the synchronized tag represents a fixed point. On the other hand, for individuals C3 and C4, the one-month tracking period shows an estimated spread of approximately 5 m, even after the Kalman filter was applied.

As shown in Fig. 2, the four individuals (C1-C4) released in October exhibited minimal movement away from their release point, designated No. 4. They remained in close proximity to this point, with average distances ranging from 1.5 m (for Individual C1) to 2.5 m (for Individual C3) from the centroid of the boulder zone at the release site. In contrast, Fig. 3 shows that most of the individuals (D1-D6) released in February exhibited significant movement away from the release point. Additionally, individuals D1, D3, and D4 were observed to remain near boulders other than those at the release site.

Quantitative evaluation of the positioning accuracy confirmed this visual impression. For the C group, the mean localization error of the synchronized tag estimated by hyperbolic positioning was 0.73 ± 0.42 m, whereas that obtained after Kalman smoothing was 0.16 ± 0.11 m (mean ± SD, *n* = 23,734). For the D group, the mean localization error estimated by hyperbolic positioning was 0.56 ± 0.30 m, whereas that obtained after Kalman smoothing was 0.006 ± 3.2 × 10⁻¹¹ m (mean ± SD, *n* = 28,079). The distribution of localization errors for each method is shown in Supplementary Fig. S1, indicating that Kalman smoothing substantially reduced the error magnitude relative to the unsmoothed estimates.

Throughout the observation period, the recorded water depth showed only small fluctuations (2.52 ± 0.097 m for the C group and 2.39 ± 0.086 m for the D group). These results indicate that tidal and diel changes in water level were minimal and had negligible influence on the stability of acoustic detection and positioning accuracy.

### Comparison of the random walk model and the UV model

**Fig. 4:**
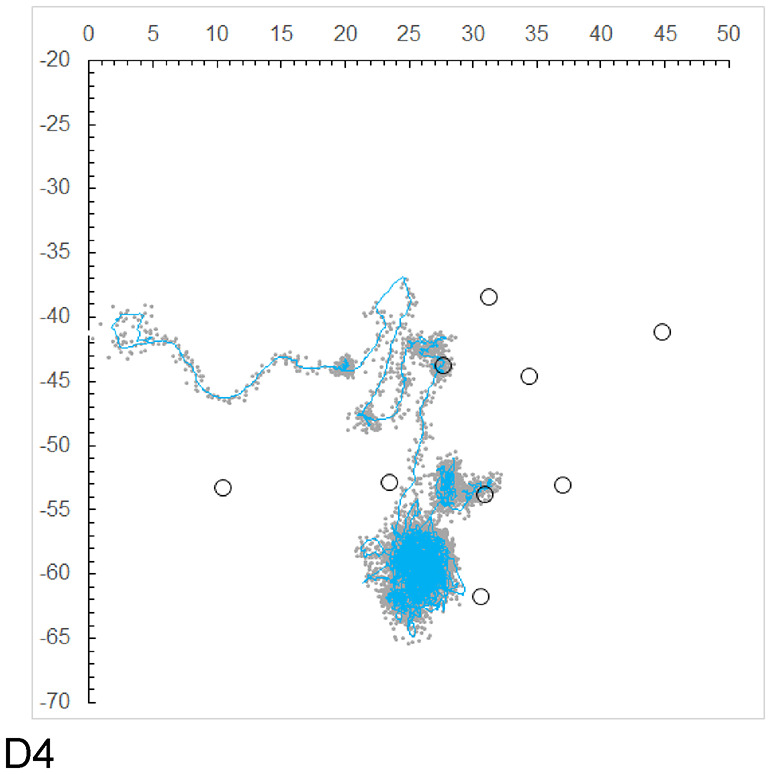
Position estimation plots ( gray dots) and movement trajectory estimation ( blue line) of Individual D4 obtained using a UV model-based Kalman filter.

To compare the movement trajectory estimated via a state estimation model based on a random walk, we apply a state estimation model that accounts for individual movement speed via equations (14) to (17). The movement trajectory of individual D4 was plotted via the Kalman filter, and the Kalman smoother was used for further refinement (Fig. 4). As shown in the figure, the movement trajectory estimated with the random walk-based state model exhibited almost no difference from that estimated with the state model incorporating movement speed.

Additionally, we calculated the movement distance and direction for each individual on the basis of the velocity vector (*u*, *v*) derived from the state estimates. The movement distance per 2-minute interval was found to be very small, at 0.002 m/(2 min) or less for all individuals. With the exception of individual D4, both the movement distance and direction remained almost unchanged over time.

### Comparison of fractal dimensions

To estimate the fractal dimension of the shapes formed by connecting the plotted points derived from the hyperbolic curves (i.e., without using the Kalman filter), we calculate the coarse-grained length *L*_*m*_(*k*) for each time series set and plot log_2_*k* versus log_2_⟨*L*(*k*)⟩. The slope of the resulting regression line was used to estimate the fractal dimension *D*.

For the calculation of the fractal dimension, we used position time series data consisting of 100 consecutive hourly estimates. However, for the synchronized tags **Csync** and **Dsync**, the fractal dimension was calculated via 200 and 140 consecutive hours of position data, respectively. Time periods with missing data exceeding one hour were excluded from the time series dataset to ensure data continuity.

**Fig. 5:**
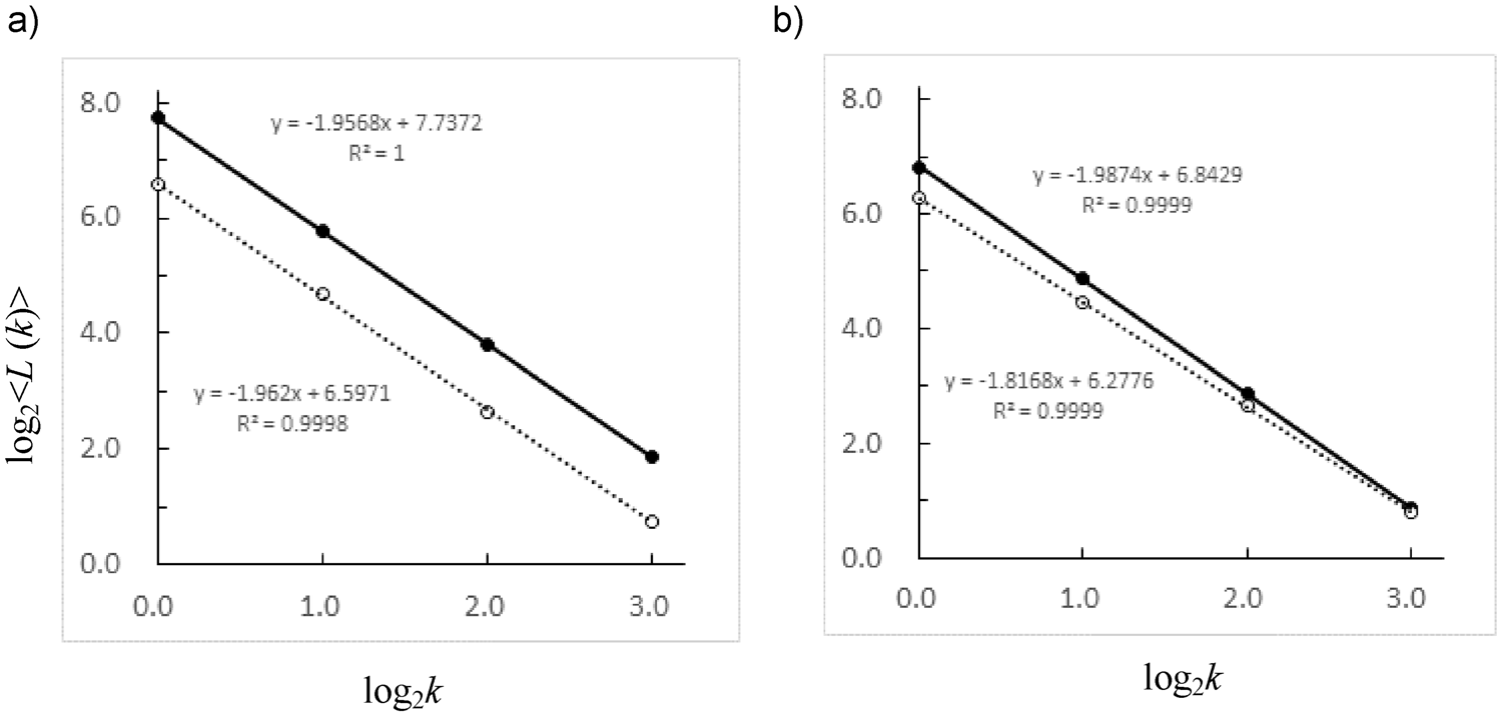
(**a**) Regression line and fractal dimension *D* calculated from the log₂k vs. log₂<*L*(*k*)> plot, based on the estimated position time series data for the synchronized tag  Csync (black circles, 200 h from October 9) and 〇 Individual C2 (white circles, 100 h from October 9). (**b**) Regression line and fractal dimension D calculated from the log₂*k* vs. log₂<*L*(*k*)> plot, based on the estimated position time series data for the synchronized tag  Dsync (black circles, 140 h from February 25) and 〇 Individual D1 (white circles, 100 h from February 26).

Figure 5 shows an example of the relationship between log_2_*k* and log_2_<*L*(*k*)>, which is calculated from the estimated positions of the fixed synchronized tags **Csync** and **Dsync**, as well as individuals **C2** and **D1**. The differences in the slopes of the regression lines for each group of plots shown in Fig. 5—representing differences in fractal dimensions—were evaluated by testing the parallelism of the regression lines via *F* values obtained from the corresponding regression analyses.

No significant difference was found in the slopes between the synchronized tag **Csync** and Individual **C2** (*F*(1,4) = 0.071, *p* = 0.80). In contrast, a significant difference was observed between the synchronized tag **Dsync** and Individual **D1** (*F*(1,4) = 115.0, *p* = 0.00043).

**Fig. 6:**
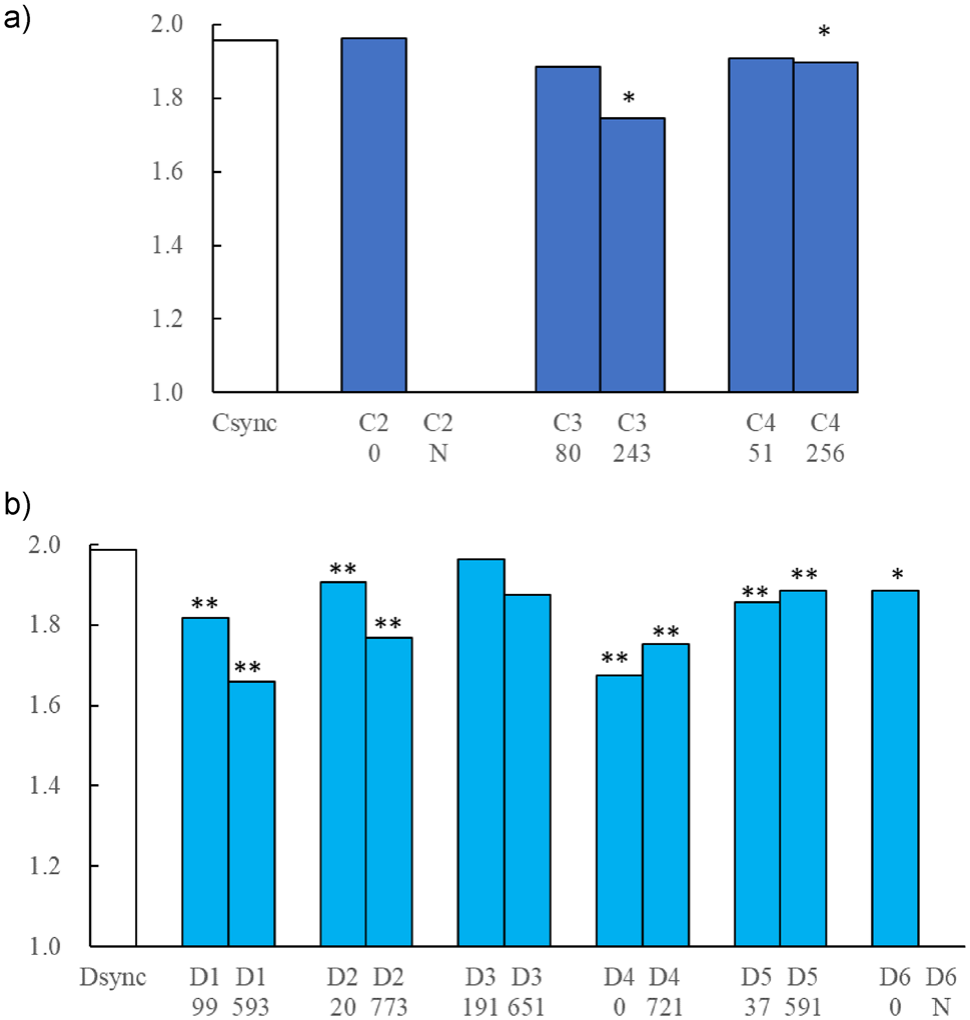
(**a**) Comparison of the fractal dimensions obtained from the time series dataset of the **C synchronized tag** plot and those of **individuals C2–C4**. (**b**) Comparison of the fractal dimensions obtained from the time series dataset of the **D synchronized tag** plot and those of **individuals D1–D6**. In both (A) and (B), the left-side bars represent data within 100 h post-release, while the right-side bars represent data from 240 h onwards. * (single asterisk) indicates significant differences at the 5% significance level, while ** **(double asterisks)** indicate significant differences at the 1% significance level, based on the test for parallelism of the regression line slopes in the log₂ *k* vs. log₂ <*L* (*k*)> plots.

The results of comparing the fractal dimensions of the synchronized tag and the tags attached to individuals **C2**, **C3**, **C4**, and **D1–D6** are shown in Fig. 6. As shown in Fig. 6a, there were no significant differences in the fractal dimensions of the estimated movement trajectories between the synchronized tag and the C individuals at the 1% significance level.

Additionally, the fractal dimension of individual **C1** could not be calculated because of an insufficient number of consecutive acoustic data points (fewer than 100). Within the first 100 h after release, no individuals presented fractal dimensions that were significantly different from those of the synchronized tag. However, after 240 h, significant differences were observed in individuals **C3** and **C4** at the 5% significance level (*F*(1,4) = 19.97, *p* = 0.011; *F*(1,4) = 18.20, *p* = 0.013). The fractal dimension of individual **C2** could not be calculated 240 h after release because of the limited availability of consecutive data.

As shown in Fig. 6b, a comparison of the fractal dimensions of the estimated movement trajectories between the synchronized tag and D individuals revealed significant differences in all individuals except **D3**. The fractal dimension of individual **D6** could not be calculated because of the insufficient number of consecutive acoustic data points obtained 240 h after release. Furthermore, a significant difference at the 5% significance level was observed between **D6** and the synchronized tag (*F*(1,4) = 9.19, *p* = 0.038).

**Fig. 7:**
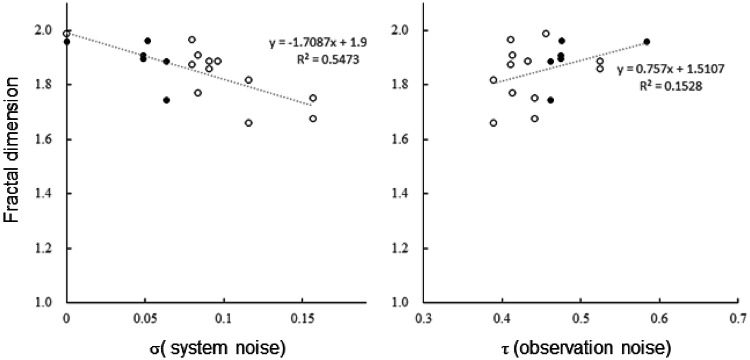
Relationship between system noise (σ) or observation noise (τ) and fractal dimension (*D*).  Black circles represent C individuals, and 〇 White circles represent D individuals. The dashed line. (---) indicates the regression line.

When the system model and observation model were represented by Eqs. (1) and (2), respectively, the standard deviation of the system noise (σ) and the standard deviation of the observation noise (τ) during the observation period for each individual were estimated via Eq. (18). The estimated values and the fractal dimensions calculated for each individual are plotted in Fig. 7 to compare their correlations. The fractal dimension tended to decrease with increasing standard deviation of the system noise σ (*R*^2^ = 0.547, *t*(16) = − 4.398, *p* < 0.001), whereas no significant correlation was observed with the standard deviation of the observation noise τ (*R*^2^ = 0.153, *t*(16) = 1.699, *p* = 0.109). Additionally, while the system noise σ clearly clustered between **C** and **D** individuals, the observation noise τ did not exhibit distinct clustering.

### Coarsening scale of movement trajectories

For slow-moving benthic organisms such as sea cucumbers, their momentum—proportional to their speed—is significantly lower than that of typical fish species, making sustained linear movement over short periods uncommon. As a result, movement trajectories, which include observational errors, tend to form more complex patterns that fill the plane, with fractal dimensions approaching **2**. The finer the segments that make up the movement trajectory (i.e., the distance traveled per unit time), the longer the total trajectory length becomes. This phenomenon is analogous to the previously mentioned **coastline paradox**, implying that the total movement distance cannot be uniquely determined.

Therefore, to explore an appropriate coarsening scale, we calculate the coarsened trajectory lengths *L*(*k*) for individuals **C** and **D**, where the coarsening scale is set as Δ*t* = *k* = 1,2,3,…,*N*_m_​ (hours). By analyzing changes in the slope − *D* of the regression line for (log_2_*k*, log_2_<*L*(*k*)>), we aimed to determine the optimal coarsening scale for the movement trajectories.

**Fig. 8:**
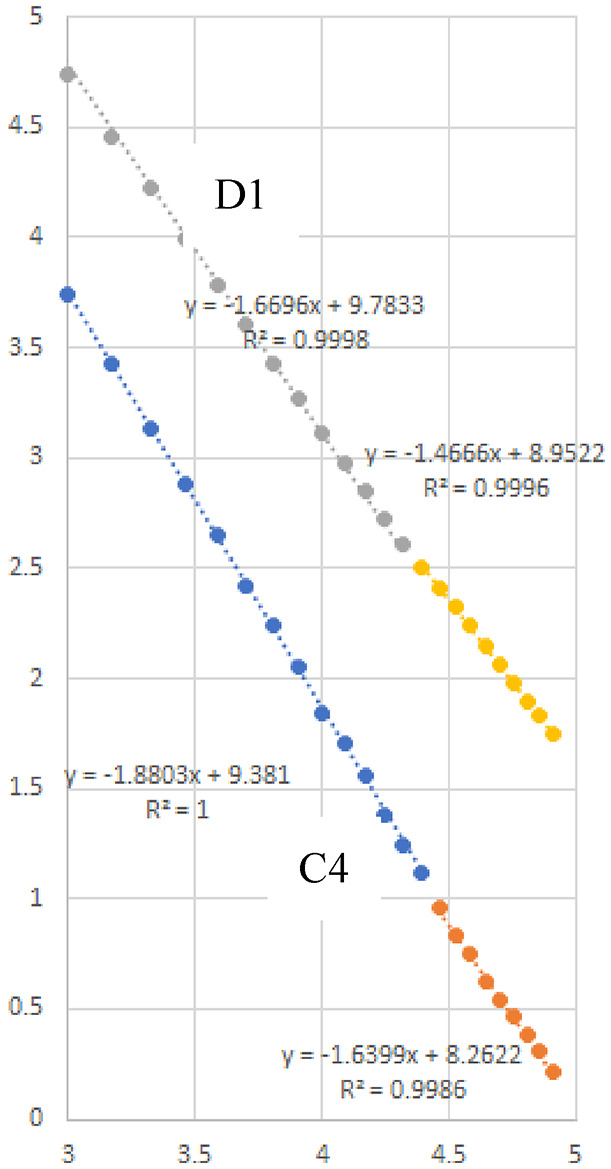
The slope of the line and the fractal dimension (*D*) of the graph of (log₂*k*, log₂<*L*(*k*)>) for individuals C4 and D1.

Figure 8 represents the slope of the linear plot of (log_2_*k*, log_*2*_<*L*(*k*)>) for individuals **C4** and **D1**. The slope of this linear plot, which reflects the **fractal dimension**, varies with coarsening scale, as shown in Fig. 8. To determine the boundary *k* at which the slope changed and to assess whether there was a significant difference in the slopes of the regression lines before and after this boundary, an ***F*** **test** was conducted via the *F* value derived from Eq. (22).

For **C** individuals, a significant change in the slope was observed for **C3** at k = 19 h (*F*(1,26) = 8.96, *p* = 0.0060) and for **C4** at k = 22 h (*F*(1,26) = 6.81 × 10, *p* = 0.0099 × 10 − 6). For **D individuals**, a significant change in the slope was observed for **D1** at k = 21 (*F*(1,26) = 2.01 × 10, *p* = 0.0013 × 10^− 1^) and for **D5** at *k* = 23 (*F*(1,26) = 1.12 × 10, *p* = 0.0025). Individual **C1** was excluded from the analysis because of a large amount of missing reception data. No significant changes in slope were observed for individuals **C2**, **D2**, **D3**, **D4**, and **D6**.

The change in the slope of (log_2_*k*, log_2_<*L*(*k*)>), i.e., the change in fractal dimension, is thought to reflect the change over time. Considering that individuals **C** and **D** may exhibit behavioral patterns with a daily cycle, the Kalman filter was applied to the estimated position plots at 12-hour intervals after release, in accordance with the sampling theorem. The 12-hour average movement speed for each individual was then calculated.

**Fig. 9:**
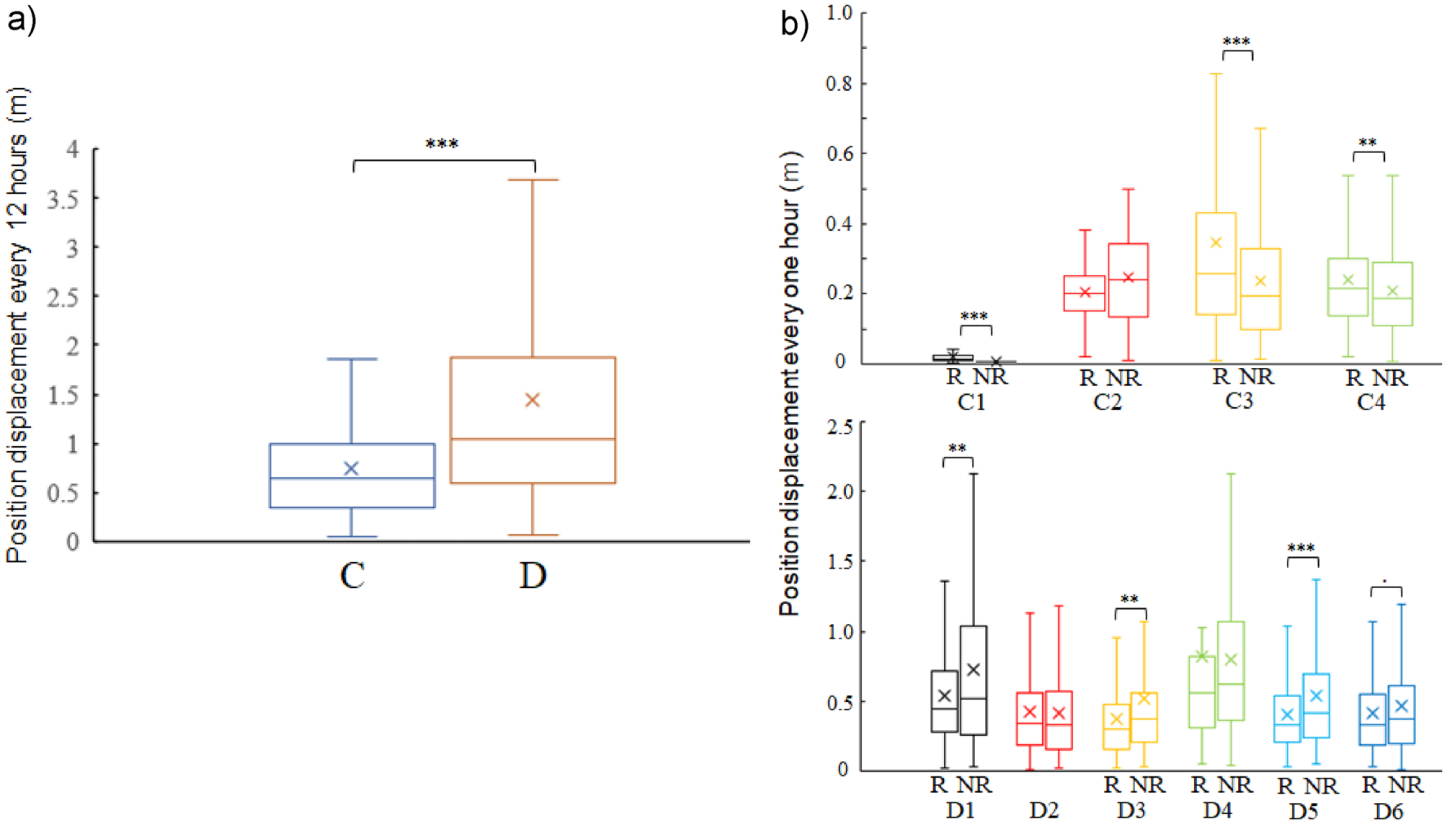
(**a**) Distance displacement by individuals C and D over 12 h (dt_1_). C individuals: 0.752 ± 0.572 m/12 h. D individuals: 1.438 ± 1.406 m/12 h. (**b**) Comparison of Distance Displacement (dt_1_) on and off Boulder Zones for Individuals C and D. The number of data points: C1(R) (*n* = 216), C1(NR) (*n* = 23), C2(R) (*n* = 104), C2(NR) (*n* = 46), C3(R) (*n* = 172), C3(NR) (*n* = 618), C4(R) (*n* = 240), C4(NR) (*n* = 550), D1(R) (*n* = 569), D1(NR) (*n* = 366), D2(R) (*n* = 603), D2(NR) (*n* = 332), D3(R) (*n* = 855), D3(NR) (*n* = 80), D4(R) (*n* = 21), D4(NR) (*n* = 914), D5(R) (*n* = 561), D5(NR) (*n* = 328), D6(R) (*n* = 580), D6(NR) (*n* = 355). Significance Levels: ***: *p* < 0.001, **: *p* < 0.01, *: *p* < 0.05, ·: *p* < 0.1 (Wilcoxon rank-sum test with continuity correction). R = Rock zone; NR = Non-rock zone.

Figure 9a shows the 12-hour average movement speeds of individuals **C1–C4** and **D1–D6** as boxplots. A comparison of the 12-hour average movement speeds revealed that **D** individuals exhibited significantly higher movement speeds (Wilcoxon rank-sum test with continuity correction, *W* = 22,896, *p* < 0.001).

Figure 9b shows boxplots comparing the hourly positional displacement (dt_1_) of individuals **C** and **D**, estimated via the Kalman filter, on the basis of whether the individuals were near the boulders or not. Proximity to the boulders was defined as being within 2 m of the centroid of the boulder zone.

The average dt_1_ during the growing stage (*D individuals*) was greater than that during the resting stage (*C individuals*), regardless of whether the individuals were near the boulders or elsewhere.

For **C** individuals during the resting stage, **C1**, **C3**, and **C4** individuals presented significantly lower average dt_1_ values **outside** the boulder zone (Wilcoxon rank-sum test, C1: *p* = 6.24 × 10⁻¹⁰; C3: *p* = 3.67 × 10⁻⁵; C4: *p* = 8.46 × 10⁻³). Conversely, for **D** individuals during the growing stage, **D1**, **D3**, and **D5** individuals presented significantly smaller average dt_1_ values near boulders (Wilcoxon rank-sum test, D1: *p* = 9.45 × 10⁻³; D3: *p* = 2.70 × 10⁻³; D5: *p* = 5.29 × 10⁻⁵).

Similarly, for **D6**, the average dt_1_ was also smaller near the boulders (*p* = 5.16 × 10⁻²).

### Relationships between individual displacement and environmental factors

Since individual D exhibited greater movement, a multiple regression analysis using a generalized linear model (GLM) was conducted in the R statistical environment (R Core Team, 2021) to investigate how the water temperature at the release point **(*X*₁)**, proximity to the boulder zone **(*X*₂)**, and day/night cycle **(*X*₃)**—defined as the time from sunrise to sunset) and at night—affect the hourly positional displacement **(dt₁)** of individuals D1–D6. The results of the analysis are presented in Table 2. The GLM analysis identified water temperature **(*X*₁)** as a significant predictor of dt₁ for all individuals, exerting a positive effect. Additionally, with the exception of individuals **D2** and **D4**, proximity to the boulder zone **(*X*₂)** had a significant suppressive effect on dt₁. The day/night variable **(*X*₃)** had a weaker influence on dt₁ than did proximity to the boulder zone; however, for individuals **D3** and **D6**, daytime was associated with a significant reduction in dt₁. Furthermore, to account for individual-level variability, we also applied a generalized linear mixed model (GLMM) including individual ID as a random effect. The GLMM results (Table 2) were consistent with the GLM findings: water temperature showed a significant positive effect on movement, whereas proximity to the boulder zone had a significant negative (suppressive) effect; the day/night cycle exhibited less consistent effects, with daytime associated with reduced movement in some individuals.

A similar analysis was conducted for individual C. In contrast to individual D, proximity to the boulder zone (X₂) had a significant positive effect on dt₁ for individuals **C1**, **C3**, and **C4**. Moreover, for individuals **C1**, **C2**, and **C3**, water temperature **(*X*₁)** was identified as a significant predictor of **dt₁** (*p* < 0.05 in all cases). Interestingly, the effect of water temperature varied: it was positive for **C2** and **C3** but negative for **C1**. The GLM analyses showed that the effects of water temperature and boulder proximity varied among C-group individuals, whereas the GLMM indicated more consistent overall patterns: boulder proximity and water temperature exerted significant positive effects (*p* < 0.01), while daytime was associated with significantly reduced movement (*p* < 0.01).

**Table 2:** Results of the GLM and GLMM analyses.

ID	Variables	Coefficients	SE	t or z value	*p* value
D1 *n* = 935	Intercept	-1.39786	0.18541	-7.539	1.12e-13 ***
	X1:Temperature (°C)	0.12515	0.02208	5.669	1.92e-08 ***
	X2: boulder zone	-0.16164	0.05513	2.932	3.45e-03 **
	X3: day/night cycle	0.06978	0.04988	1.399	1.62e-01
D2 *n* = 935	Intercept	-1.83615	0.18089	-10.151	< 2e-16 ***
	X1:Temperature (°C)	0.12657	0.02196	5.765	1.11e-08 ***
	X2: boulder zone	0.04104	0.05605	0.732	4.64e-01
	X3: day/night cycle	-0.07814	0.05261	-1.485	1.38e-01
D3 *n* = 934	Intercept	-1.14643	0.19158	-5.984	3.10e-09 ***
	X1:Temperature (°C)	0.08776	0.02200	3.989	7.15e-05 ***
	X2: boulder zone	-0.42796	0.09600	-4.458	9.28e-06 ***
	X3: day/night cycle	-0.20222	0.05392	-3.751	1.87e-04 ***
D4 *n* = 934	Intercept	-1.16086	0.15791	-7.352	4.29e-13 ***
	X1:Temperature (°C)	0.11726	0.02058	5.698	1.63e-08 ***
	X2: boulder zone	0.03693	0.17321	0.213	8.31e-01
	X3: day/night cycle	0.04312	0.04995	0.863	3.88e-01
D5 *n* = 888	Intercept	-1.72968	0.18570	-9.314	< 2e-16 ***
	X1:Temperature (°C)	0.13655	0.02306	5.922	4.54e-09 ***
	X2: boulder zone	-0.12367	0.05359	-2.308	2.13e-02 *
	X3: day/night cycle	-0.03005	0.04997	-0.601	5.48e-01
D6 *n* = 934	Intercept	-1.50480	0.17241	-8.728	< 2e-16 ***
	X1:Temperature (°C)	0.10490	0.02134	4.915	1.05e-06 ***
	X2: boulder zone	-0.13889	0.05346	-2.598	9.52e-03 **
	X3: day/night cycle	-0.11022	0.05142	-2.144	3.23e-02 *
GLMM for D *n* = 5560	Intercept	-1.48138	0.11193	-13.235	< 2e-16 ***
	X1:Temperature (°C)	0.11548	0.00834	13.846	< 2e-16 ***
	X2: boulder zone	-0.13287	0.02447	-5.430	5.64e-08 ***
	X3: day/night cycle	-0.05199	0.02026	-2.566	1.03e-02 *

For the GLM, coefficients, standard errors, t values, and p values of the explanatory variables are shown for each D individual. For the GLMM, coefficients, standard errors, z values, and p values are presented, with individual ID treated as a random effect. Significance codes: *** *p* < 0.001, ** *p* < 0.01, * *p* < 0.05.

## Discussion

### Tracking and movement estimation

In this study, the movement of *A. japonicus* was estimated through tracking with acoustic tags, enabling the assessment of behavioral patterns in slow-moving benthic organisms. When the movement of such slow-moving animals is measured via acoustic tags, the relative proportion of measurement errors increases compared with the actual movement, making accurate estimation of movement trajectories challenging. However, this issue can be mitigated by applying the Kalman filter.

The acoustic tags used in this study were attached using a base similar in design to spaghetti tags. During the experimental period in February, five out of the six individuals retained the acoustic tags (V5-1 H, VEMCO) for up to 40 days, indicating a high retention rate. This finding supports the results of Furukawa et al. who reported higher tag retention rates during low-temperature winter periods than during high-temperature summer periods^8^. On the basis of these results, the attachment method used in this study is sufficiently effective for investigating movement characteristics when acoustic tags of this size are employed.

In cases where experimental or measurement system errors can be estimated in advance and there is a clear basis for determining the noise terms, these errors are often estimated beforehand. This approach is common in engineering system design. Examples include the measurement of angular velocity and angular acceleration via extended Kalman filters in sports, as well as tracking methods for badminton shuttles that utilize motion blur^41^.

In contrast, when tracking animal behavior—especially for species with wide-ranging movements—preestimating the noise terms within a state‒space model (SSM) is challenging. As a result, the noise terms are often treated as unknown parameters and estimated via maximum likelihood estimation (MLE) on the basis of observational data^33^. Both MLE and Markov chain Monte Carlo (MCMC) methods are commonly applied for noise estimation in SSMs.

For example, Anderson-Sprecher and Ledolter modeled the movement of mule deer (*Odocoileus hemionus*) as a two-dimensional random walk and estimated system and observation model errors within the SSM via MLE^42^. Similarly, Sibert et al. estimated the horizontal movement trajectories of bigeye tuna near Hawaii via a Kalman filter with a random walk system model^29^. Nielsen et al. applied an extended Kalman filter to account for nonlinearities in geolocation models that incorporated light levels and sea surface temperature (SST) when tracking tagged blue sharks (*Prionace glauca*)^43^.

For MCMC-based approaches, Jonsen et al. modeled the movements of leatherback turtles (*Dermochelys coriacea*) in two-dimensional space, estimating nonlinear system noise within the SSM as an unknown parameter from behavioral observation data^44,45^. In this study, we treated the noise components in the SSM as unknown parameters and estimated them numerically on the basis of the collected data. Since our SSM is linear and assumes Gaussian noise, we were able to derive these estimates relatively easily via numerical optimization algorithms applied to the likelihood function. However, if (*u*, *v*) represents the long-term drift of slow-moving benthic organisms such as this species, it may provide insight into the directional trend of individual movement.

The resulting state‒space model, which incorporates the estimated noise parameters, effectively represents the movement of *A. japonicus*. Hammond reported that the movements of the chocolate chip sea cucumber (*Isostichopus badionotus*) and the donkey dung sea cucumber (*Holothuria mexicana*) in homogeneous environments could be characterized as random walks^46^. In system models that account for movement velocities (*u*, *v*), these components can be interpreted as time-varying drift terms added to the position coordinates. While such drift components have been incorporated into system models for species such as bigeye tuna and blue sharks^29,43^, our results suggest that the drift velocities (*u*, *v*) are minimal and have little impact on trajectory estimation. Therefore, a simpler system model that does not explicitly incorporate these drift terms is sufficient, making the analysis more straightforward.

In the present study, the seafloor was largely flat with small boulders no higher than 0.5 m, and all individuals were observed either on the sandy bottom or within spaces between boulders. Therefore, vertical displacement on the seafloor was negligible relative to the positioning uncertainty (~ 2 m). However, in environments characterized by large rocks or steep bottom relief, such vertical components should be considered when evaluating movement energetics and dispersal patterns.

In this study, the standard deviations of system noise (σ) and observation noise (τ) were treated as unknown parameters and estimated via MLE. When a random walk system model was applied, the standard deviation of the observation noise for stationary synchronized tags was 0.58 m in 2020 and 0.46 m in 2021. The corresponding system noise (σ) was estimated to be 2.4 × 10⁻⁶ m in 2020 and 4.1 × 10⁻⁷ m in 2021. These values for observation noise are of the same order of magnitude as expectations on the basis of the speed of underwater acoustic propagation, whereas the extremely small system noise values confirm the appropriateness of the model for stationary tags.

In addition, during both the C-group and D-group experiments, fixed synchronized transmitters were deployed within the receiver array, and their positions were estimated via hyperbolic positioning and after Kalman filter smoothing (Figs. 2 and 3). The dispersion of the unsmoothed positions around the known fixed locations provided an empirical estimate of local positioning error, which was consistent with the theoretical spatial resolution derived from the receiver clock resolution (1/1000 s) and sound speed. Although this assessment was limited to the fixed synchronized tag sites, the C-group individuals remained predominantly near the release point, and the D-group individuals did not move outside the receiver array. Therefore, it is unlikely that positioning accuracy deteriorated relative to the fixed synchronized tag locations.

This conclusion was further supported by our new quantitative comparison (Supplementary Fig. S1), which showed that the Kalman-smoothed positions exhibited substantially smaller localization errors than the unsmoothed hyperbolic estimates. Depth records obtained during the observation period showed only minor fluctuations, indicating that tidal and diel water-level changes had negligible influence on positioning accuracy.

The experimental site was a fishing port where strong wave and tidal influences were minimal, with a generally flat sandy bottom interspersed with multiple boulders, resulting in limited potential for acoustic shadowing. While spatial error in acoustic positioning can vary in more complex or noisy environments, under the present fishing port conditions such effects were likely negligible.

Using the random walk model, we successfully reconstructed the movement trajectories of *A. japonicus*. This modeling approach proved effective not only during periods of low activity, such as resting stage but also during the more active winter months, demonstrating its versatility for different behavioral states.

### Evaluation of nocturnal behavior on the basis of movement speed

Purcell and Kirby developed an individual-based movement model (IBM) for the sandfish (*Holothuria scabra*) using field data on speed and directional distribution, as well as the relationship between speed and body mass, to determine the appropriate size of no-take zones (NTZs)^47^. In this study, we compared movement speed and directional angular displacement. The estimated movement distance per hour, derived from body weight via an experimental regression equation, was expressed as:

$${\text{speed }}({\text{cm }}{{\text{h}}^{{\text{-1}}}})\,=\,0.0{\text{4237}} \times {\text{mass }}\left( {\text{g}} \right)$$

This estimate closely matched the values obtained in our study. However, since nighttime measurements were not conducted, the overall diel movement pattern remains unclear, and many juveniles have been reported to remain inactive during the daytime.

Additionally, Dong et al. conducted tank experiments to measure the emergence rate of *A. japonicus* juveniles from shelters at different times of day. Their results revealed a relatively high emergence rate at night, indicating a cyclic nocturnal activity rhythm in *A. japonicus* juveniles under all tested photoperiod conditions^25^. Furthermore, Masaki et al. reported an increased emergence rate from stone piles at night in an indoor behavioral observation experiment using juvenile *A. japonicus*^17^.

In this study, the daytime movement speed significantly decreased in two individuals during the early growing stage in February and in one individual during the resting stage period. Although the data did not strongly indicate nocturnal behavior, it is possible that the individuals emerged from beneath or between rocks at night.

Sun et al. reported that *A. japonicus* exhibits more active feeding and movement behavior at night than during the day, even in complete darkness. These findings suggest that, in the short term, diel feeding and locomotion behavior are regulated by endogenous rhythms rather than external factors such as light or low temperatures^20^.

In this study, we conducted a generalized linear model (GLM) analysis to examine the relationships between movement distance per unit time and sunrise/sunset as categorical variables. However, no strong correlation was detected between diel phases and movement distance. While the relationship between illumination and movement remains unclear, movement behavior may have been influenced by the time of day.

However, diel behavioral patterns can vary by species and developmental stage. Hammond reported that adult *I. badionotus* and *H. mexicana* presented increased nocturnal activity, whereas the sand sea cucumber (*Holothuria arenicola*) presented no significant variation in the feeding rate over a 24-hour cycle^46^. These findings suggest that species-specific and ontogenetic differences may influence diel activity rhythms.

### Comparison of movement speeds with previous studies

According to Mercier et al., the sea cucumber *H. scabra* exhibited a daily movement distance of approximately 1.3 m under natural conditions^48^. Similarly, Purcell et al. reported that the leopard sea cucumber (*Bohadschia argus*) moved an average of 1.9 m day^− 1^ in Palfrey Lagoon, with an average body weight of 1.3 kg—significantly greater than that of the *A. japonicus* specimens examined in the present study^23^.

In this study, during the growing stage, individuals whose wet weight ranged from 191 to 257 g experienced an average movement of 1.438 m over a 12-hour period, which exceeds the values reported in these previous studies. It is possible that more detailed telemetry-based analyses, such as those conducted in this study, could reveal greater movement distances in these species.

On the other hand, Hamel et al. reported that the sea cucumber *C. frondosa* relies on semibuoyant movement mechanisms^22^, allowing it to achieve movement speeds significantly greater than those of benthic crawling. Their study demonstrated that this species could tumble along the seafloor with the current, reaching a maximum speed of 54.7 ± 26.3 cm s^− 1^. While such current-assisted movement was not observed in this study, similar behavior may be detected in areas outside fishing ports where currents are stronger.

### Relationships among the flow distribution, resting location, water temperature, and movement

Sun et al. reported that when *C. frondosa* individuals were introduced into experimental tanks with varying flow velocity distributions, more than half of them remained in regions with flow speeds below 20 cm s^− 1^ after 96 hours^49^.

In the present study, we did not analyze the relationship between the flow velocity distribution within the fishing port and individual movement. However, the presence of boulders may create flow shadows within the port, potentially influencing individuals’ preferences for certain resting locations. Our previous study revealed that *A. japonicus* near boulders remained stationary during rough weather, whereas others were displaced by waves^50^.

In the present study, as shown in Fig. 9b, individuals exhibited slower movement speeds near boulders during the growing stage. This suggests that they might have been utilizing the flow shadows created by the boulders. Interestingly, Fig. 9b also shows that during less active periods, such as October (the *resting stage*), movement speeds were higher near boulders, whereas during active periods, such as February (the *growing stage*), movement speeds were lower near boulders. This implies that boulders may serve as resting spots during active periods.

The results of the GLM analysis indicated that during the early recovery phase (*growth stage*), proximity to the boulder zone significantly affected movement speed. All four individuals analyzed (D1, D3, D5, and D6) exhibited reduced movement speeds near the boulder zone. In contrast, during the resting stage phase, proximity to the boulder zone significantly increased movement speed, with all three analyzed individuals (C1, C3, and C4) exhibiting increased movement speeds near boulders. Furthermore, the application of a generalized linear mixed model (GLMM), which incorporated individual ID as a random effect, confirmed the robustness of these results. The GLMM supported the GLM findings by showing that proximity to the boulder zone suppressed movement during the growing stage but enhanced movement during the resting stage.

With respect to the relationship between water temperature and movement speed, during the *growing stage*, higher water temperatures significantly increased movement speeds for all individuals. In the GLM analysis at the individual level, no clear relationship between water temperature and movement was detected during the resting stage; however, the GLMM analysis revealed that, similar to the growing stage, water temperature exerted a positive effect on movement during the resting stage as well.

Kato and Hirata reported that *A. japonicus* exhibited active behavior at low temperatures ranging from 12 to 17 °C, with activity peaking at 16°C^51^. They also reported that activity was greater at night and during the day as temperatures decreased. In the present study, the average water temperature during the *growing stage* in February was 10.7 °C (minimum 4.2 °C, maximum 10.7 °C), whereas in October (*resting stage*), it was 16.4 °C (minimum 11.3 °C, maximum 20.2 °C). In contrast to the findings of Kato and Hirata, movement in the present study was sluggish at temperatures considered optimal for activity. Their experiment used adult *A. japonicus* collected from southern Japanese waters, where the environmental conditions differ significantly from those in our study area, potentially explaining the observed behavioral differences.

Sun et al. reported that the feeding activity of *A. japonicus* peaked at 16 °C, whereas locomotor behavior was unaffected by water temperatures below 24°C^20^. In the present study, 16 °C was the average water temperature during the resting stage; however, individuals wandered near boulders without significant movement. Although the water temperature gradually decreased during the resting stage phase, the lack of a clear relationship between temperature and movement supports the findings of Sun et al. ^20^ regarding locomotor behavior.

### The coastline paradox and fractal dimensions

Ru et al. measured the locomotion speed of *A. japonicus* collected from Rushan Bay across different growth stages (176–310 g) in a tank environment^52^. Using time-lapse video, they calculated the daily movement distances, reporting 32.04 ± 2.77 m per day for the smallest individuals and 15.82 ± 1.14 m for those in later growth stages. These values are considerably larger than the daily movement distances observed in this study.

While this discrepancy may partially reflect differences between tank and field conditions, another plausible explanation lies in the coastline paradox—where finer and more continuous measurements of movement trajectories result in substantially greater estimated distances.

In this study, we calculated the fractal dimensions of two-dimensional trajectory plots representing the time series positions of *A. japonicus*. By comparing the fractal dimensions of fixed synchronized tags with those of tags attached to individuals, we observed no significant differences during the resting stage period in October. However, during the growing stage in February, significant differences emerged in many individuals.

Furthermore, the fractal dimension was negatively correlated with system noise in the state‒space model (Fig. 7). This suggests that fractal dimensions can serve as a simple metric to compare the degree of randomness in individual movements. Additionally, fractal dimension analysis may be a useful method for directly evaluating raw time series data before preprocessing with Kalman filtering.

Nonetheless, system noise in the state‒space model may provide a more precise representation of movement randomness than fractal dimensions do. This is because system noise estimated via the Kalman filter clearly separates individual movement characteristics into distinct seasonal clusters. Furthermore, by distinguishing between observational noise and system noise in positional data, the state-space model offers a more accurate and substantive representation of the fundamental movement characteristics of individuals.

### Effectiveness of the proposed method

Masaki et al. attempted to evaluate the effectiveness of releasing artificially cultured *A. japonicus* by investigating the factors contributing to the decline in detection rates of individuals released on artificial reefs^4,17^. Their approach relied on direct underwater observations to assess movement and dispersal characteristics. However, tracking the movement patterns of individual sea cucumbers via this method is extremely challenging.

In this study, we successfully achieved long-term tracking of individual sea cucumbers by equipping them with lightweight transmitters that minimized the gravitational load. Furthermore, by applying the Kalman filter with a system model based on a random walk, we were able to achieve highly accurate movement tracking. The observation of movement patterns throughout the year allows us to capture seasonal changes in the movement characteristics of sea cucumbers within the port. Additionally, in the future, it will be possible to assess the effects of hydrodynamic conditions on movement by incorporating current meters and wave gauges.

While this study was conducted in a hydrodynamically sheltered environment, several cited works (e.g., Eriksson & Byrne, Barkai, Hamel et al., Purcell et al.) investigated holothurian behavior in more energetic open-coast settings^15,21–23^. Behavioral characteristics in such environments may differ from those observed here. Nevertheless, in the absence of extreme bathymetric gradients, the analytical framework developed in this study could also be applied to open-coast habitats. Future research should aim to quantify the influence of wave action and tidal currents on sea cucumber behavior through the deployment of wave gauges and current meters, enabling more detailed analyses of the relationship between hydrodynamic conditions and movement patterns. We plan to address this in upcoming experiments.The results suggest that boulders influenced movement patterns. In the current state‒space model, movement trajectories were effectively represented via a simple random walk model without velocity terms. However, if we incorporate behavioral models that account for environmental factors such as sea cucumber preferences for boulders and the influence of water flow on movement speed, the system model could be further refined. This would enable the estimation of unknown parameters related to boulder preference and environmental influence on the basis of observational data.

Lillacci and Khammash successfully estimated parameters related to the heat response process in *Escherichia coli* via an extended Kalman filter^53^. Similarly, by using advanced state estimation techniques such as the ensemble Kalman filter, it would be possible to develop a more detailed behavioral model. This, in turn, could lead to strategies for controlling sea cucumber movement through the strategic placement of boulders.

Appropriate placement of boulders may help retain released sea cucumbers within designated port areas. In the near future, we aim to construct a more detailed behavioral model for *A. japonicus* and report our findings.

In the present study, we found that environmental factors such as water temperature, the presence of boulders, and diel phase clearly influenced movement distances of *A. japonicus* within a sheltered environment. These behavioral responses are relevant to stock enhancement programs, as retaining individuals within release areas during critical periods—such as the growing stage in February, when feeding requirements are high—may reduce the risk of displacement and enhance post-release survival. Our findings suggest that boulders can function as physical structures that help maintain individuals within target areas, while higher water temperatures may increase movement potential during both growing and resting stages. Although empirical demographic data such as survival or reproductive success were not available in the present study, future integration of such data with movement analyses could clarify how behavioral patterns influence population dynamics in released stocks.

## Supplementary Information

Below is the link to the electronic supplementary material.

Supplementary Material 1

## Data Availability

The datasets used for the production of this paper are not publicly available but may be made available by the corresponding author upon request for reasonable reasons.
