# Supplementary material for: Data assimilation reveals behavioral dynamics of sea cucumbers as a model for slow-moving benthic animals
Source: Sci Rep. 2025 Dec 12;16:77. doi: 10.1038/s41598-025-29171-3 (PMC12764805; doi:10.1038/s41598-025-29171-3)
Supplement: Supplementary file 1 — Supplementary Material 1 [file 41598_2025_29171_MOESM1_ESM.docx]

**Supplementary Information**

**Data Assimilation Reveals Behavioral Dynamics of Sea Cucumbers as a Model for Slow-Moving Benthic Animals**

**Tsutomu Takagi^1*^, Yuto Tanaka^2^, Erica Sasano^1,4^, Kouki Kanda^1,5^, and Yuichi Sakai^3^**

1 Faculty of Fisheries Sciences, Hokkaido University, Sapporo 060-0810, Japan.

2 Graduate School of Env. Science, Hokkaido University, Sapporo 060-0810, Japan.

3 Hakodate Fisheries Research Institute, Hakodate 040-0051, Japan.

4 Present address: Kansai Electric Power Co., Inc., Osaka 530-8270, Japan.

5 Present address: Organo Co., Inc., Tokyo 136-8631, Japan.

*** Corresponding author: Tsutomu Takagi, email:tutakagi@fish.hokudai.ac.jp**

| **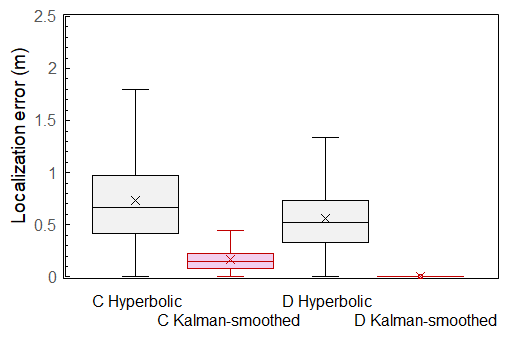** |
| --- |
| **Supplementary Figure S1.** Distribution of localization errors (horizontal distance between the true location measured by a laser 3D rangefinder and the estimated positions) for hyperbolic positioning and after Kalman smoothing.Boxes indicate the interquartile range, horizontal lines represent the median, and whiskers show the data range. The fixed synchronized tag for the C group was observed for 34 days, whereas that for the D group was observed for 40 days. |
